# Supplementary figures and images for: Xbp1 and Brachyury establish an evolutionarily conserved subcircuit of the notochord gene regulatory network
Source: eLife. 2022 Jan 20;11:e73992. doi: 10.7554/eLife.73992 (PMC8803312; doi:10.7554/eLife.73992)

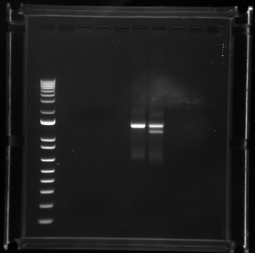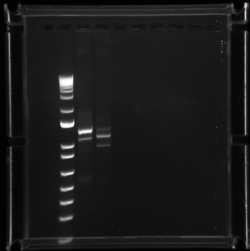

Supplement: Figure 5—figure supplement 1—source data 1. [file elife-73992-fig5-figsupp1-data1.pdf]

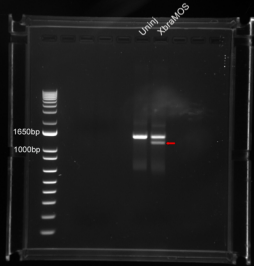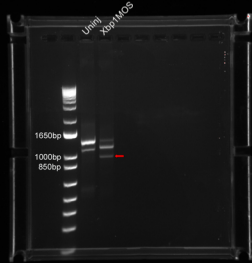

Supplement: Figure 5—figure supplement 1—source data 2. [file elife-73992-fig5-figsupp1-data2.pdf]
