# Supplementary material for: Xbp1 and Brachyury establish an evolutionarily conserved subcircuit of the notochord gene regulatory network
Source: eLife. 2022 Jan 20;11:e73992. doi: 10.7554/eLife.73992 (PMC8803312; doi:10.7554/eLife.73992)
Supplement: Transparent reporting form [file elife-73992-transrepform1.pdf]

Westbrook Centre
Milton Road
Cambridge CB4 1YG, UK
P 01223 855340
W elifesciences.org
T @elife

eLife's transparent reporting form

We encourage authors to provide detailed information *within their submission* to facilitate the interpretation and replication of experiments. Authors can upload supporting documentation to indicate the use of appropriate reporting guidelines for health-related research (see EQUATOR Network), life science research (see the BioSharing Information Resource), or the ARRIVE guidelines for reporting work involving animal research. Where applicable, authors should refer to any relevant reporting standards documents in this form.

If you have any questions, please consult our Journal Policies and/or contact us: editorial@elifesciences.org.

Sample-size estimation

- You should state whether an appropriate sample size was computed when the study was being designed
- You should state the statistical method of sample size computation and any required assumptions
- If no explicit power analysis was used, you should describe how you decided what sample (replicate) size (number) to use

Please outline where this information can be found within the submission (e.g., sections or figure legends), or explain why this information doesn't apply to your submission:

The number of *Ciona* transgenic embryos analysed and used to prepare graphs is reported in each graph (Fig. 2C, Fig. 4G). Biological replicates were obtained for each experiment; a biological replicate is defined here as an experiment carried out using the same amounts of DNA and the same electroporation conditions on a different batch of animals. Additional information and references are included in the material and methods section (page 19 and following pages).
The number of *Xenopus* embryos analysed was determined based on robustness of phenotypes across multiple biological replicates (different clutch of embryos obtained from different females). This information is included in the material and methods section (page 22 and following pages).

Replicates

- You should report how often each experiment was performed
- You should include a definition of biological versus technical replication
- The data obtained should be provided and sufficient information should be provided to indicate the number of independent biological and/or technical replicates
- If you encountered any outliers, you should describe how these were handled
- Criteria for exclusion/inclusion of data should be clearly stated
- High-throughput sequence data should be uploaded before submission, with a private link for reviewers provided (these are available from both GEO and ArrayExpress)

Please outline where this information can be found within the submission (e.g., sections or figure legends), or explain why this information doesn't apply to your submission:

Only probe sets with *p*-values ≤0.05 with an absolute fold change cut-off of 2.0 were further considered. The complete dataset has been deposited into the NCBI Gene Expression Omnibus (GEO), under accession number GSE46751. Additional details can be found on

eLife Sciences Publications, Ltd is a limited liability non-profit non-stock corporation incorporated in the State of Delaware, USA, with company number 5030732, and is registered in the UK with company number FC030576 and branch number BR015634 at the address Westbrook Centre, Milton Road, Cambridge CB4 1YG.

page 22 of the manuscript.

The probes used for *Ciona* whole-mount in situ hybridizations were synthesised from the available collections of ESTs, and are referenced and listed in each panel (Fig. 3 and Fig. S2), along with their respective gene models.

Over 30 *Xenopus* embryos per condition were used, and experiments were repeated on at least 3 different biological replicates (different clutch of embryos obtained from different females). This information is included in the material and methods section (page 23). Information about the total number of embryos analysed for each injection/treatment can be found in the graphs providing quantification of the phenotypes (n=; see Fig 5 and Fig 6). For in-situ hybridization experiments only embryos with co-localized expression of the lineage tracer with the cell type marker were considered for analysis. This information is included in the material and methods section (page 23).

Statistical reporting

- Statistical analysis methods should be described and justified
- Raw data should be presented in figures whenever informative to do so (typically when N per group is less than 10)
- For each experiment, you should identify the statistical tests used, exact values of N, definitions of center, methods of multiple test correction, and dispersion and precision measures (e.g., mean, median, SD, SEM, confidence intervals; and, for the major substantive results, a measure of effect size (e.g., Pearson's r, Cohen's d)
- Report exact p-values wherever possible alongside the summary statistics and 95% confidence intervals. These should be reported for all key questions and not only when the p-value is less than 0.05.

Please outline where this information can be found within the submission (e.g., sections or figure legends), or explain why this information doesn't apply to your submission:

(For large datasets, or papers with a very large number of statistical tests, you may upload a single table file with tests, Ns, etc., with reference to sections in the manuscript.)

Group allocation

- Indicate how samples were allocated into experimental groups (in the case of clinical studies, please specify allocation to treatment method); if randomization was used, please also state if restricted randomization was applied
- Indicate if masking was used during group allocation, data collection and/or data analysis

Please outline where this information can be found within the submission (e.g., sections or figure legends), or explain why this information doesn't apply to your submission:

eLife Sciences Publications, Ltd is a limited liability non-profit non-stock corporation incorporated in the State of Delaware, USA, with company number 5030732, and is registered in the UK with company number FC030576 and branch number BR015634 at the address Westbrook Centre, Milton Road, Cambridge CB4 1YG.

Additional data files ("source data")

- We encourage you to upload relevant additional data files, such as numerical data that are represented as a graph in a figure, or as a summary table
- Where provided, these should be in the most useful format, and they can be uploaded as "Source data" files linked to a main figure or table
- Include model definition files including the full list of parameters used
- Include code used for data analysis (e.g., R, MatLab)
- Avoid stating that data files are "available upon request"

Please indicate the figures or tables for which source data files have been provided:

> Two source data files have been provided for Fig. S3, in the form of an image of the full raw unedited gel and an image of the uncropped gel with the relevant bands clearly labeled.
